# AG1478 inhibits the migration and invasion of cisplatin-resistant human lung adenocarcinoma cells via the cell cycle regulation by matrix metalloproteinase-9

**DOI:** 10.3892/ol.2014.2224

**Published:** 2014-06-04

**Authors:** LI MA, HUIQIN YAN, QINHUA ZHOU

**Affiliations:** 1 Department of Medical Oncology, Tianjin Medical University General Hospital, Tianjin 300050, P.R. China; 2 Tianjin Key Laboratory of Lung Cancer Metastasis and Tumor Microenviroment, Tianjin Lung Cancer Institute, Tianjin Medical University General Hospital, Tianjin 300052, P.R. China

**Keywords:** AG1478, cisplatin resistance, metastasis, cell cycle, matrix metalloproteinase-9

## Abstract

AG1478 is a specific epidermal growth factor receptor (EGFR) tyrosine kinase inhibitor. The effect of AG1478 on the A549/DDP (cisplatin-resistant human lung adenocarcinoma) cell line is unknown. The aim of the present study was to investigate the effects of AG1478 on the A549/DDP cell line and its sensitive parental A549 cell line. The two cell lines were treated with AG1478 and the growth, proliferation, migration and invasion of the tumor cell lines were measured using flow cytometry, as well as 3-(4,5-dimethylthiazol-2-yl)-2,5-diphenyltetrazolium bromide, wound healing and Transwell system assays. The expression of metastasis-associated genes and proteins was evaluated by quantitative-polymerase chain reaction and western blot analysis. The molecular mechanisms were investigated using short-interfering RNAs (siRNAs). The phosphorylation status of the key cell cycle protein, retinoblastoma (Rb), was also investigated. The results revealed that AG1478 inhibited the growth of the two cell lines with varying potency, and that the A549/DDP cell line was more sensitive to AG1478 than the A549 cell line. Cell migration and invasion, as well as matrix metalloproteinase (MMP)-9 and E2F1 expression were significantly inhibited. However, MMP-9 expression was also significantly suppressed in the two cell lines following transfection with E2F1-targeting siRNA. In addition, AG1478 significantly arrested A549/DDP and A549 cells in G_1_ phase, with a corresponding reduction in the S phase. The phosphorylation of Rb protein at various sites was selectively inhibited by AG1478 at various time points. The results indicate that AG1478 may provide a clinical therapeutic approach for certain types of cisplatin-resistant lung cancer.

## Introduction

Lung cancer is the leading cause of cancer-associated mortality worldwide. Chemotherapy is the predominant treatment for lung cancer, which may improve patient survival and quality of life, particularly in advanced cases (1). Cisplatin is one of the cytotoxic agents used in clinical chemotherapy. However, the therapeutic effects of cisplatin are limited due to intrinsic or acquired drug resistance. Anticancer drugs used in chemotherapy may increase the acquired resistance of tumor cells. This increased resistance enhances tumor metastasis, which further increases their drug resistance (2,3). At present, none of the available treatment regimens are capable of preventing the metastasis of drug-resistant tumor cells. Epidermal growth factor receptor (EGFR) has been found to correlate with key characteristics of cancer, including cell proliferation, apoptosis and tumor metastasis (4,5), and the dysregulation of EGFR has been associated with chemoresistance in lung cancer (6,7). Gefitinib and erlotinib are EGFR-tyrosine kinase inhibitors (TKIs) that have been approved for lung cancer treatment (8). Clinical studies have shown that these EGFR-TKIs were effective in patients who had been treated previously with multiple cytotoxic agents, however, no significant effects were identified in patients who had not received chemotherapy (9–12). AG1478 is a quinazoline with a similar chemical structure and mechanism of action as erlotinib and gefitinib (13,14). To determine whether AG1478 inhibits A549/DDP cell growth, migration and invasion *in vitro*, the antitumor mechanism of AG1478 in the A549/DDP and A549 cell lines was investigated.

## Materials and methods

### Reagents

AG1478 was purchased from Merck KGaA (Darmstadt, Germany). The rabbit polyclonal antibody against matrix metalloproteinase (MMP)-9, the retinoblastoma (Rb) antibody sampler kit, including the phosphor-Rb antibodies Ser780, Ser795 and Ser807, as well as the total Rb mouse monoclonal antibody and the rabbit monoclonal antibody against GAPDH (14C10) were purchased from Cell Signaling Technology, Inc. (Danvers, MA, USA). The horseradish peroxidase-conjugated affinipure goat anti-rabbit IgG (H+L) and goat anti-mouse IgG (H+L) secondary antibodies were purchased from ZSGB-BIO (Beijing, China) and the reverse transcription and quantitative polymerase chain reaction (qPCR) kits were purchased from Takara Biotechnology (Dalian) Co., Ltd., (Dalian, China). The rabbit polyclonal antibody against E2F1 was purchased from Santa Cruz Biotechnology, Inc. (Santa Cruz, CA, USA). The E2F1 short-interfering RNA (siRNA) and HiPerFect transfection reagent were purchased from Qiagen (Hilden, Germany), and 3-(4,5-dimethylthiazol-2-yl)-2,5-diphenyltetrazolium bromide (MTT) was purchased from Invitrogen Life Technologies (Carlsbad, CA, USA).

### Cell lines

The cisplatin-resistant A549/DDP and cisplatin-sensitive A549 cell lines were provided by the Tianjin Lung Cancer Institute (Tianjin, China). Cells were cultured and maintained in RPMI-1640 medium supplemented with 10% fetal bovine serum (FBS) and 2 mmol/l glutamine (both Gibco-BRL, Grand Island, NY, USA) at 37°C in a humidified atmosphere of 5% CO_2_.

### Cell proliferation assay

Cells were cultured in 96-well plates (8,000 cells/well) overnight and treated with dimethyl sulfoxide (DMSO) as the control or AG1478 for 48 h. The effects of AG1478 on the proliferation of the A549/DDP and A549 cell lines were measured using the MTT assay, as previously described (15). The MTT solution (5 mg/ml) was added to the cell cultures and incubated for 4 h at 37°C. The cell suspensions were treated with DMSO and subjected to colorimetric measurement at a wavelength of 570 nm using the TriStar LB 941 apparatus (Berthold Technologies U.K. Ltd., Harpenden, UK). DMSO was used for the blank absorbance readings. The rate of cell growth inhibition and IC_50_ were calculated using the GraphPad Prism 4 software (GraphPad Software, Inc., La Jolla, CA, USA).

### Wound healing assay

The cells were seeded in six-well plates to 100% confluence. A wound was induced by scratching the cell cultures with a pipette tip. Following rinsing with phosphate-buffered saline (PBS) to remove the detached cells, AG1478 (at near IC_50_ concentration) was added to culture in a 5% CO_2_ incubator for 48 h at 37°C. The cells were incubated and allowed to migrate in the medium. Images were immediately captured from each well, and again after 48 h using a TE2000 inverted fluorescence microscope (Nikon Corporation, Tokyo, Japan) in four random fields at ×40 magnification. The width of the wound at these specific locations was visualized on each plate to quantify the rate of cell migration.

### Cell invasion assay

Cell invasion assays were performed using 24-well Transwell plates (8 mm pore size; Corning Inc., Acton, MA, USA) coated with 1 mg/ml Matrigel (BD Biociences, Franklin Lakes, NJ, USA). A total of 1.0×10^5^ cells/well were suspended in 300 μl of serum-free media and added to the upper compartment of the Transwell plates. Next, 500 μl complete media containing 10% FBS was added to the bottom wells of each plate. The cells were then incubated in a 5% CO_2_ incubator, with or without AG1478 (at near IC_50_ concentration), for 48 h at 37°C. Invasive and non-invasive cells on the upper and lower surface of the membrane were stained according to the manufacturer’s instructions. Non-invasive cells retained in the upper chamber were removed with a cotton swab and the invasive cells were examined using a TE2000 inverted fluorescence microscope in ph1 mode (Nikon Corporation).

### Cell cycle analysis

Cell lines were treated with AG1478 (at near IC_50_ concentration) for 48 h. Cells were then collected by trypsinization and fixed with 70% ethanol by incubating them overnight at 4°C in the dark. The cell pellets were then resuspended in PBS and stained with propidium iodide/RNase staining buffer (BD Biociences) for 30 min at 37°C. Analysis was performed using a FACS Aria flow cytometer (Becton Dickenson, Franklin Lakes, NJ, USA), and the cell cycle data were processed using the ModFit LT cell cycle analysis software (Verity Software House, Topsham, ME, USA).

### qPCR analysis

The total RNA was extracted from the cells using TRIzol (Invitrogen Life Technologies). Reverse transcription was performed using a DNA Engine Peltier Thermal Cycler (Bio-Rad, Hercules, CA, USA) using a reverse transcription kit (Takara Biotechnology (Dalian) Co., Ltd.) according to the manufacturer’s instructions, as previously described (16). Standard qPCR was performed using the following primers: Forward, 5′-CATCCCAGGAGGTCACTTCTG-3′ and reverse, 5′-GACAACAGCGGTTCTTGCTC-3′ for E2F1; forward, 5′-GGGACGCAGACATCGTCATC-3′ and reverse, 5′-TCGTCATCGTCGAAATGGGC-3′ for MMP-9; and forward, 5′-GGAGTCAACGGATTTGGTCG-3′ and reverse, 5′-CTTGATTTTGGAGGGATCTCG-3′ for GAPDH. All primers were synthesized by the Bejing Genomics Institute (Shenzhen, China). mRNA levels were detected by qPCR using SYBR Green stain. The PCR reaction conditions used were as previously described (16).

### Western blot analysis

Following treatment with or without AG1478 (at near IC_50_ concentration) for 24 and 48 h, the A549/DDP and A549 cells were lysed in pre-warmed Laemmli buffer (Bio-Rad). Western blot analysis was performed as previously described (17). Briefly, the same amount of total protein from each sample was resolved by SDS-PAGE on a well of 10% polyacrylamide gel (Amersham Pharmacia Biotech, Piscataway, NJ, USA) and resolved by SDS-PAGE. The phosphor-Rb (Ser780, Ser795 and Ser807) and total Rb antibodies were used at 1:500 dilutions, whereas all the other primary antibodies (E2F1, MMP-9 and GAPDH) were used at 1:750 dilutions. Protein expression was quantified by densitometry using the Transparency Adapter for PowerLook 2100XL (UTA-2100XL; UMAX, Mountain View, CA, USA).

### E2F1 siRNA transfection

The E2F1 siRNA-1 (5′-CAGGACCTTCGTAGCATTGCA-3′) and siRNA-2 (5′-ACGCTATGAGACCTCACTGAA-3′) were transfected using the HiPerFect transfection reagent (Qiagen), according to the manufacturer’s instructions. Cells were transfected for 24 or 48 h and washed twice with cold PBS. The cell pellets were subsequently collected to determine their E2F1 and MMP-9 expression levels using qPCR and western blot analysis, as described above.

### Statistical analysis

Student’s t-test was used to determine the significance of the differences between the control and experimental groups. Error bars were used to indicate the standard deviation of the data and P<0.05 was considered to indicate a statistically significant difference.

## Results

### AG1478 inhibits cell proliferation

**Figure 1:**
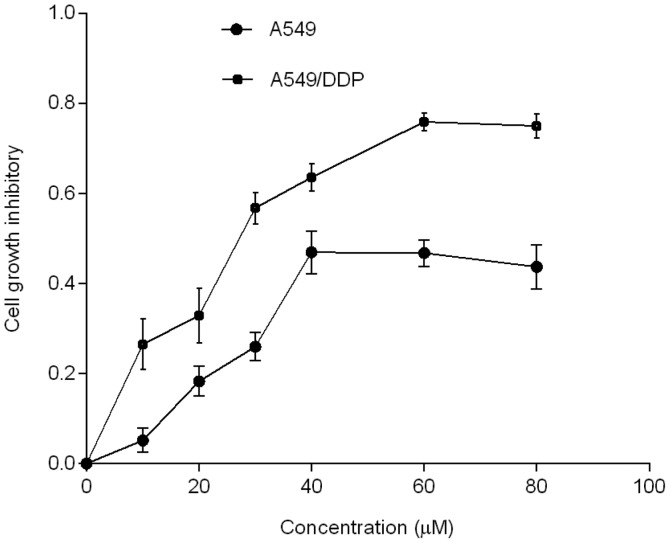
Effect of AG1478 on tumor cell growth in the A549/DDP and A549 cell lines. Data are presented as the mean ± standard deviation from three independent measurements.

The results indicated that AG1478 inhibited the growth of the two cell lines with varying potency. The IC_50_ values of AG1478 in the cisplatin-resistant A549/DDP cell line (33.6±3.45 μM) were lower than those of the corresponding parental A549 cell line (65.6±5.92 μM), as shown in Fig. 1.

### AG1478 arrests cells at G_1_ phase

**Figure 2:**
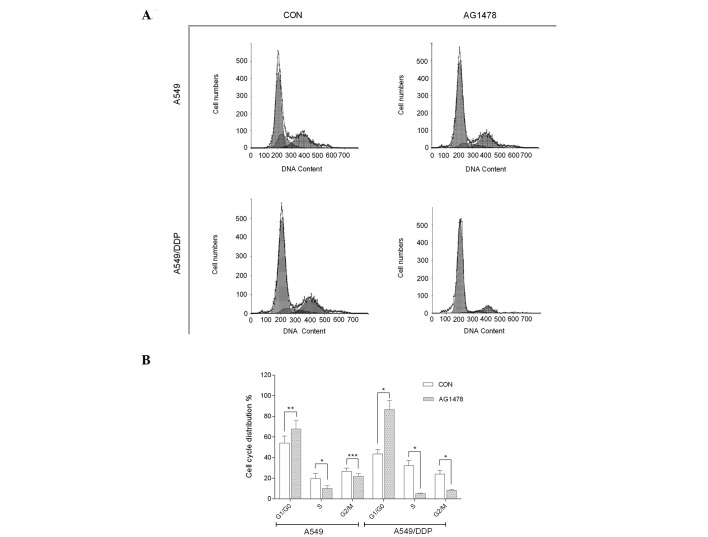
Effect of AG1478 on tumor cell cycle. (A) Cell cycle profiles of the control- and AG1478-treated A549/DDP and A549 cell lines. (B) Cell cycle distribution of the control- and AG1478-treated A549/DDP and A549 cell lines. P-values were determined using Student’s t-test. (^*^P<0.001, ^**^P<0.05 and ^***^P>0.05).

To further examine whether AG1478 inhibits cell proliferation, the percentage cell distribution at various stages of the cell cycle was determined in the two cell lines based on their DNA content by flow cytometry. The results showed that AG1478 significantly inhibited DNA synthesis in the treated cells, when compared with the untreated cells (P<0.001). In addition, FACS analysis revealed that the untreated proliferating A549/DDP cells exhibited the following cell cycle distributions: 43.5±4.50% in G_1_/G_0_ phase; 32.2±5.21% in S phase; and 24.3±3.53% in G_2_/M phase. However, the parental A549 cells were composed of the following cell cycle distributions: 54.0±6.91% in G_1_/G_0_ phase; 18.9±5.01% in S phase; and 26.7±3.22% in G_2_/M phase. By contrast, the treated proliferative A549/DDP cells exhibited the following cell cycle distribution: 86.6±8.91% in G_1_/G_0_ phase; 5.19±0.52% in S phase; and 8.22±0.92% in G_2_/M phase. However, the treated A549 cells exhibited the following distributions: 67.8±8.42% in G_1_/G_0_ phase; 10.1±3.03% in S phase; and 22.1±2.52% in G_2_/M phase. AG1478 significantly arrested A549/DDP cells in G_1_ phase (P<0.001), with a corresponding reduction in the S and G_2_/M phases (P<0.001). AG1478 similarly blocked the A549 cells from progressing beyond the G_1_ phase (P<0.05), with a simultaneous reduction in the S phase (P<0.001). However, a significant reduction in the G_2_/M phase was not observed in the A549 cells (P>0.05), as shown in Fig. 2.

### AG1478 inhibits cell migration and invasion

**Figure 3:**
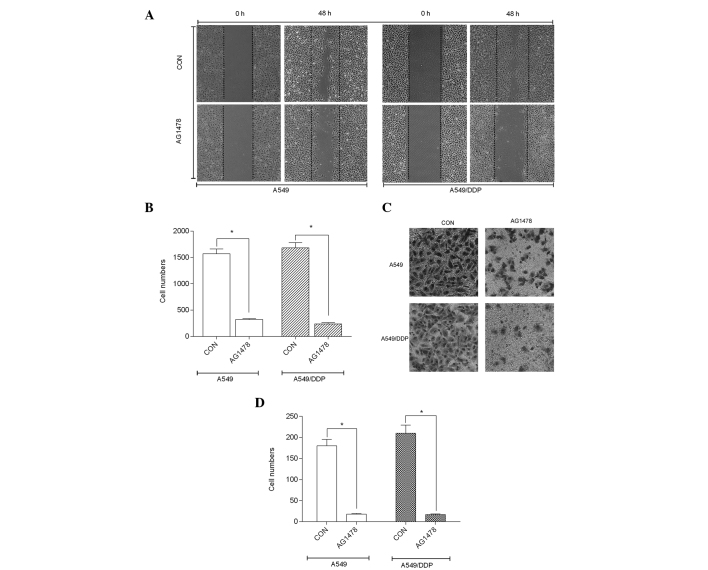
Effect of AG1478 on tumor cell migration and invasion of control- and AG1478-treated A549/DDP and A549 cell lines. (A) Migration image and (B) analysis of the migration image results. (C) Invasion image and (D) analysis of the invasion image results. The P-values were determined using the Student’s t-test (^*^P<0.001).

To investigate the effect of AG1478 on migration and invasion, the two cell lines were treated with AG1478 (almost IC_50_, respectively) for 48 h, whereas the controls were left untreated. Cell migration was analyzed using the wound-healing assay, as described above. Wounds generated on the AG1478-treated cells did not heal for 48 h, whereas wounds in the untreated cell lines had almost completely healed, as shown in Fig. 3A. Furthermore, the migration levels of the A549/DDP and A549 cells were reduced to 15.4±1.21 and 13.6±1.68%, respectively, after 48 h (P 0.001), as shown in Fig. 3B. AG1478 inhibited the migration of the two cell lines, as confirmed by quantitative analysis using a Transwell system. The mean invasive proportion of the AG1478-treated cell lines was reduced to 10.1±1.31% in the A549/DDP cells (P<0.001) and 8.7±0.63% in the A549 cells (P<0.001), as compared with the control, as shown in Fig. 3C and D.

### AG1478 regulates MMP-9 expression via E2F1

**Figure 4:**
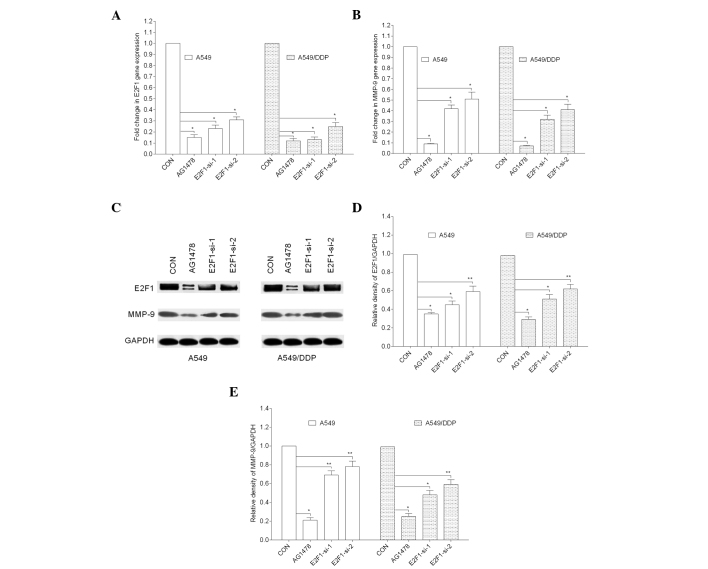
Regulation of AG1478 on MMP-9 and E2F1 in the control-, AG1478- and E2F1-targeting siRNA-treated A549/DDP and A549 cell lines. Gene expression of (A) E2F1 and (B) MMP-9. (C) Western blot analysis of E2F1 and MMP-9 expression. Analysis of (D) E2F1 and (E) MMP-9 protein levels in the two cell lines. P-values between control, and AG1478-treated or E2F1-targeting siRNA were determined using Student’s t-test. (^*^P<0.001 and ^**^P<0.05). siRNA, small-interfering RNA.

E2F1 is a transcriptional activator of MMP-9 that regulates lung cancer cell invasion and metastasis (18). The present study showed that AG1478 almost completely eliminated MMP-9 and E2F1 gene expression (P<0.001). To further determine whether AG1478 modulates the expression of MMP-9 in A549/DDP and A549 cells via the E2F1 transcription factor, the two cell lines were transfected with 10 nM siRNA against E2F1 or with a non-targeting control siRNA. MMP-9 gene and protein expression following transfection with E2F1-targeting siRNA were significantly reduced in the two cell lines (P<0.001 and P<0.05), as shown in Fig. 4.

### AG1478 modulates Rb protein status

**Figure 5:**
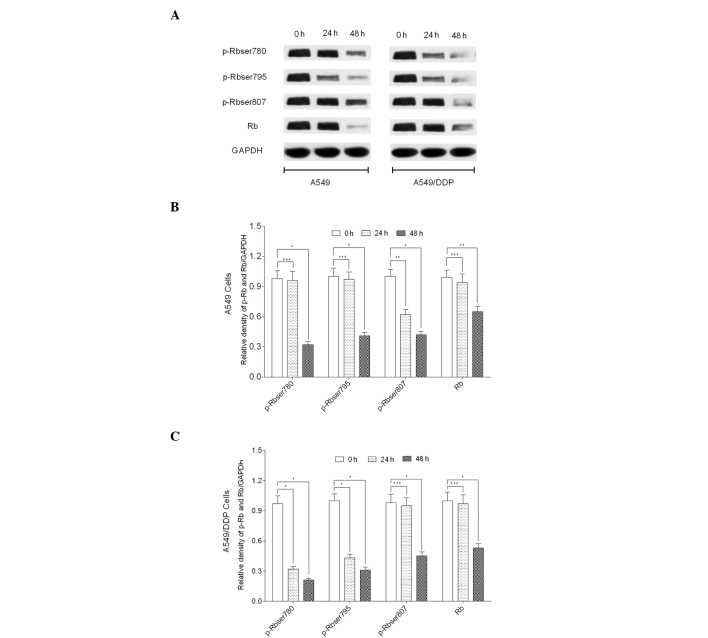
Effect of AG1478 on phosphorylation status of Rb. (A) Western blot analysis. Rb protein levels in (B) A549/DDP and (C) A549 cell lines. P-values between 0 and 24 h, and 0 and 48 h were determined using Student’s t-test (^*^P<0.001, ^**^P<0.05 and ^***^P>0.05). Rb, retinoblastoma.

To further determine whether AG1478 modulates the key cell cycle protein, Rb, the phosphorylation status of Rb protein was determined. The results revealed that AG1478 significantly inhibited the phosphorylation of Rb at Ser780 and Ser795 sites following the exposure of A549/DDP cells to AG1478 for 24 h (P<0.001). However, no significant reduction in the phosphorylation at the Ser807 site was identified (P>0.05). By contrast, AG4178 significantly inhibited the phosphorylation of Rb at Ser807 sites in A549 cells following 24 h exposure (P<0.05); however, the Ser780 and Ser795 sites were not affected (P>0.05). Furthermore, a significant reduction in Rb phosphorylation was identified at Ser780, Ser795 and Ser807 sites of A549/DDP (P<0.001) and A549 (P<0.05) cell lines at 48 h (Fig. 5).

## Discussion

The chemoresistance of cancer cells is a major obstacle in the treatment of malignant cancers. The enhanced sensitivity of cancer cells to chemotherapy is highly desirable. The present study demonstrated that the A549/DDP cell line was more sensitive to AG1478 than the A549 cell line. Similar results have been observed in two other cisplatin resistant oral squamous carcinoma cell lines, with an increased sensitivity to the novel EGFR inhibitor AG1478 (19). As a cytotoxic chemotherapeutic agent, cisplatin causes DNA damage and may arrest cells in the G_2_ phase (20,21). The current study revealed that AG1478 blocked the two cell lines in the G_1_ phase of the cell cycle, with a concomitant decrease in the proportion of cells in S phase, which caused cell cycle redistribution. Rb is a key cell cycle protein, which inhibits entry into S phase during the cell cycle. Rb functions together with the E2F-family of transcription factors to activate or inhibit cell proliferation (22). As one of the activating transcription factors, E2F1 promotes cell cycle progression into S phase when Rb is inactivated by phosphorylation (23), and E2F1 functions as an activator of MMPs to modulate MMP-9 expression (18). The present study found that AG1478 inhibited E2F1 and MMP-9 expression, and reduced the levels of E2F1 via RNA interference, which consequently decreased the expression of MMP-9. Cell cycle progression usually occurs when Rb is inactivated by phosphorylation, leading to the release of free E2F1 (24). This phenomenon facilitates the expression of E2F1 target genes and promotes cell proliferation. The current study revealed that AG1478 selectively inhibited the phosphorylation of Rb, thereby facilitating its activation in various sites at different time points in the two cell lines, and consequently eliminated the expression of E2F1. These results suggest that Rb may be activated by AG1478 via dephosphorylation, thereby preventing the release of free activating E2F1. This may consequently inhibit the expression of target genes, such as MMP-9, preventing the progression of the cell cycle and subsequently leading to the suppressed tumor cell migration and invasion observed. This study may provide a promising therapeutic approach for a particular type of cisplatin-resistant lung cancer.
